# Percutaneous nephrolithotomy for 1-2 cm lower-pole renal calculi

**DOI:** 10.4103/0970-1591.44264

**Published:** 2008

**Authors:** Percy Jal Chibber

**Affiliations:** Department of Urology, Jaslok Hospital and Research Centre, 15, G Deshmukh Road, Mumbai-400 026, India

**Keywords:** Complications, lower calyx, management, percutaneous nephrolithotomy, renal calculi, technique

## Abstract

**Objectives:**

The most appropriate management of patients with lower-pole calyceal (LC) stones remains controversial. In this review we discuss the role of percutaneous nephrolithotomy (PCNL) in the management of LC stones 1–2 cm in maximum dimension.

**Materials and Methods:**

A detailed literature review was performed to summarize the recent technical developments and controversies in PCNL. The results of PCNL for 1-2 cm LC calculi were reviewed.

**Results:**

PCNL is increasingly employed as a primary modality in the treatment of LC calculi. It has a high success rate and acceptably low percentage of major complications in experienced hands. Supine position is found to be as safe and effective as prone position. Urologist-acquired access is associated with fewer access-related complications and better stone-free rates. Ultrasound is increasingly employed as an imaging modality for obtaining access. There have been increasing reports of tubeless PCNL in the literature. Most patients undergoing tubeless PCNL do not need hemostatic agents as an adjuvant for hemostasis. Non-contrast computed tomography does not yield statistically valuable increase in the diagnosis of significant residual stones compared with that of plain X-ray and linear tomography. Comprehensive metabolic evaluation and aggressive medical management can control new stone recurrences and growth of residual fragments following PCNL.

**Conclusions:**

PCNL is a highly effective procedure with consistently high stone-free rates when compared with extracorporeal shockwave lithotripsy or retrograde intrarenal surgery. The results also do not depend on anatomic factors and stone size. It is associated with low morbidity in experienced hands.

## INTRODUCTION

The most appropriate management of patients with lower-pole calyceal (LC) stones remains controversial. The preferred approaches are extracorporeal shockwave lithotripsy (SWL) for stones < 1 cm and percutaneous nephrolithotomy (PCNL) for those > 2 cm.[1] For stones 1–2 cm in size, there is a decline in the use of SWL with a parallel increase in use of PCNL and retrograde intrarenal surgery (RIRS), since they are associated with better stone-free rates.[1–3]

After >30 years of worldwide experience, PCNL remains a milestone technique in the field of endourology with a high success rate and acceptably low percentage of major complications.[4] The success of PCNL for treatment of LC calculi dose not depend on the anatomic factors that usually affect the outcome of SWL and RIRS.[5] It is also almost independent of stone size.[6,7] Larger the stone the more efficient is its percutaneous removal. In this review we discuss the recent technical development in PCNL and its role in the management of LC stones 1–2 cm in dimension.

## METHODOLOGY OF REVIEW

Pub-med search was performed in June 2008 using the terms “lower calyx, renal calculi, PCNL, complications, management, technique, percutaneous access.” Titles and/or abstracts were reviewed to determine relevance to this article. Only articles discussing the recent technical developments in PCNL and its role in the management of LC stones 1–2 cm in dimension were included in this review.

## INDICATIONS

Indications of treating LC calculi are the same as those for stones located in other locations and include increasing size, localized obstruction, associated infection, hematuria and chronic or acute pain.[6] Asymptomatic renal stones larger than 1 cm in size are associated with a 47% risk of developing a symptomatic episode within two years if left untreated. Hence, prophylactic therapy is advisable for stones > 1 cm in size.[6] The only absolute contraindications for PCNL are untreated coagulopathy and pyonephrosis.

## TECHNICAL CONSIDERATIONS

### Position: prone vs. supine

The prone position is accepted globally due to its familiarity, excellent understanding of the anatomy in this position, and reduced risk of visceral complications. However, the supine position is preferable particularly in morbidly obese patients and in those with cardiorespiratory compromise and stature deformity. It is found to be as safe and effective as the prone position. The major technical disadvantage of the supine position is in accessing the upper calyx.[8,9]

### Imaging: fluoroscopy vs. ultrasound

Access to the pyelocaliceal system is routinely performed using fluoroscopic guidance. However, there is an increasing use of ultrasound for gaining access during PCNL. Till date, no survey has been undertaken to know the percentage of centers that prefer ultrasound for obtaining percutaneous access. It is proposed that ultrasonography provides a real time three-dimensional monitoring of the puncture, thereby minimizing the chances of segmental artery injury and decreasing the blood loss during the procedure.[10] In a randomized control trial Basiri *et al*., found ultrasoundguided access an acceptable alternative to fluoroscopy guidance and was associated with lower radiation exposure. Its success and complication rate were comparable to those of fluoroscopy-guided PCNL.[11]

### Access: Urologist vs. Radiologist

The success and complications of PCNL seem to be directly related to the ability to achieve optimal access. A survey from the US conducted in 2003 showed that only 11% of urologists performing PCNL routinely obtain percutaneous access themselves.[12] The reason for this trend may include lack of training, comfort level and perceived need of radiological involvement. However, urologists are increasingly obtaining access themselves because this eliminates reliance on a second “surgeon” and increases flexibility with respect to procedure timing and the location of the access tract.[13] Watterson *et al*., in a retrospective study comparing urologist- vs. radiologist-acquired access found that access-related complications were less and stone-free rates were improved during urologist-acquired percutaneous access.[14]

### Access: site and number

**Figure 1:**
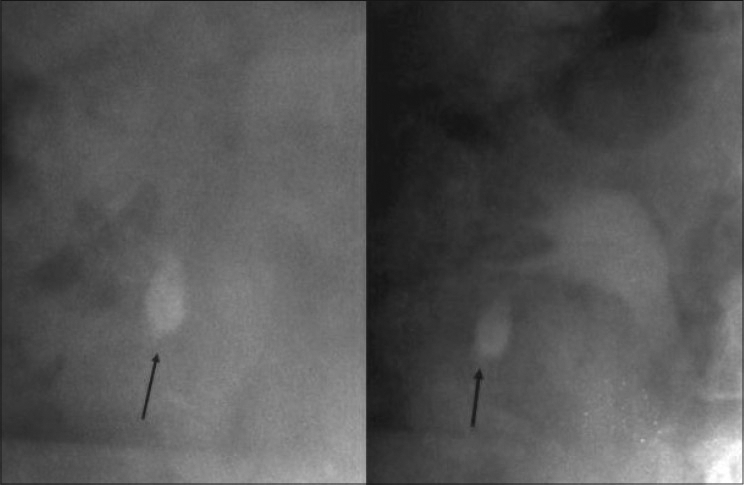
Complex lower-pole calculi: May need access to two separate calices. Upper pole access not possible

**Figure 2:**
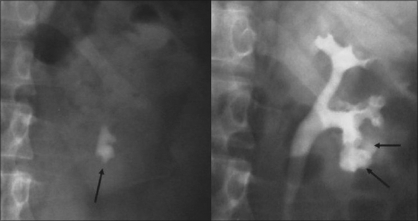
Bilateral renal calculi with spastic pelvicaliceal system. Will need bilateral multiple punctures

**Figure 3:**
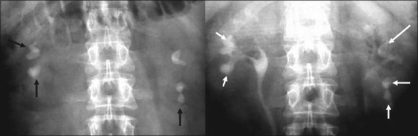
Single LC calculus with narrow indfundibulum: Lower pole access

The successful removal of stones requires accurate placement of percutaneous tract that provides direct access to the stone. Inferior calyceal stones are usually approached through the inferior calyx [Figure 1]. However, in complex inferior calyceal calculi, complete clearance may not be possible through a single tract in the inferior calyx because of problems in negotiating the acute angles between the calyces[15] [Figures 2 and 3]. Aron *et al*., compared the outcome of upper pole access vs. lower pole access for treating complex lower-pole calculi. They found that upper pole access provided faster and better stone clearance with a single puncture, and was associated with less requirement of a second-look procedure.[15]

Multiple percutaneous tracts (Y- tract) might be required in some patients with complex LC calculi. This aggressive approach is highly effective in achieving stone clearance but at the cost of increased blood loss.[8,10,16] On the contrary, Hegarty *et al*., found that the blood loss and complication rates in PCNL with multiple tracts are comparable to those of PCNL incorporating a single percutaneous tract. They found a significant rise in serum creatinine and drop in creatinine clearance in patients needing multiple tracts.[17]

### Post-PCNL drainage

Routine placement of nephrostomy tube after an uncomplicated PCNL is being seriously questioned. Since its initial description in 1997, there have been increasing reports of tubeless PCNL in the literature.[18] In this, the percutaneous nephrostomy is replaced by indwelling ureteral stent or a ureteric catheter at the end of an uncomplicated PCNL.[9,18] It is based on the principle that simple closure of tract with a dressing or parietal suture creates a closed retroperitoneal compartment, which is ideal for achieving self-tamponade. This corresponds to a clamped nephrostomy tube.[19] There are also a few case series on totally tubeless and stentless PCNL in properly selected patients.[20]

To minimize or eliminate the risk of bleeding or extravasation after tubeless PCNL, a few authors have employed hemostatic agents in the nephrostomy tracts as an adjuvant to PCNL.[21,22] Borin *et al*., describe using haemostatic gelatin matrix (FloSeal; Baxter Inc., Irvine, CA) to provide hemostasis of the tract after tubeless PCNL. The authors occluded the collecting system at the level of entry of the Amplatz sheath with an occlusion balloon catheter, passed retrograde. FloSeal was then injected through the partially retracted Amplatz sheath while withdrawing the applicator and the sheath in tandem. The guide wire was withdrawn per urethra until its tip resided in the renal pelvis. A 36-cm, 7F tail stent was passed retrograde, and the skin closed with cyanoacrylate adhesive (Ethicon, Somerville, NJ).[22] However, in a randomized control trial employing haemostatic fibrin sealant Tisseel™ after tubeless PCNL it was noted that instillation of haemostatic agents did not decrease postoperative bleeding or hemorrhagic complications but only resulted in less postoperative pain and a marginal decrease in hospital stay. The authors felt that most patients undergoing tubeless PCNL do not need these haemostatic agents and its associated cost.

## ASSESSMENT OF STONE-FREE STATUS

Although most urologists agree that the goal of PCNL is to achieve stone-free status, the determination of stone-free status varies according to the diagnostic tool used. Historically, plain radiography was accepted as the standard method to judge residual stones following stone surgery. But recently, non-contrast computed tomography (NCCT) has proved to be the most sensitive tool for detecting residual stones after PCNL. The sensitivity for detection of residual fragments was 47.6% for plain radiographs films as judged by NCCT.[23] In spite of this, all the articles published on the efficacy of PCNL for LC calculi have not employed NCCT scan to determine stone-free status [Table 1]. NCCT may yield false positive results. There is a possibility of “over reading” with the rate reaching 15% after a secondary operation with flexible nephroscopy.[24] A recent study also recommended that it should not be routinely performed in patients with opaque stones since it yields no statistically valuable increase in the diagnosis of significant residual stones compared with that of plain X-ray and linear tomography.[25]

**Table 1:** Summary of published literature on percutaneous nephrolithotomy for LC calculi management

Author/Year	N	Study type	Stone size	SFR	Complications	Comments
McDougal, 1989[7]	29	RCS with SWL	1-2 cm	66.6%	-	First study comparing outcome of PCNL vs. SWL for LC calculi.
				86.2%		Higher SFR with PCNL than SWL. (86.2 % vs. 54.2 %)
Netto NR, 1991[28]	23*	RCS with SWL	1.42 cm	93.3%	20%	Recurrence - 13% at 18 months
	15			95.6%*	56.52%*	
	(1-2 cm size)				urosepsis-8.7% BT-4.3	PCNL is associated with statistically significant SFR than SWL
Lingeman JE, 1994[7]	32* 11 (1-2 cm)	CS & metaanalysis	1-2 cm	100%	4- UTI, 2-pleural effusion, 1- bleeding without BT	Stone recurrence 22% at 12.1 + 8.8 months In meta-analysis for 1-2 cm size stone, higher SFR for PCNL than SWL (89% vs. 56%)
Havel D, 1998[31]	73	RCS with SWL	1-2	72.5%	-	SFR for PCNL statistically better than SWL (72.5% vs. 44%) but with higher morbidity
Albala DM, 2001[30]	58*	Multicentric	< 3 cm	92%*	22%	
	29 (1-2 cm)	prospective RCT of SWL vs. PCNL			22% 1-UTI, 3- ileus, 1-sepsis, 2-hematoma, 1-obstruction, 3- perforation, 1- BT, 1- AV fistula	Calculi > 1 cm are better managed by PCNL than SWL PCNL offers higher SFR than SWL (95 % vs. 37%)
Ziaee S, 2004[39]	45	CS	<2.5 cm	88%	No major	PCNL morbidity low if performed by skilled person
Aron M, 2004[15]	102*	RCS of upper pole vs. lower pole access for LC calculi	896.8 mm^2^*	84.3%	12.74%	Superior pole access offers better clearance through a single puncture (87% vs. 79% SFR) and less need for re-look procedure (3% vs. 18%)
Nowak K, 2005#[32]	175*	RCS with SWL	1-2 cm	76%	-^#^	PCNL is more effective then SWL especially for stones > 1 cm.
Staios D, 2007[40]	22**	CS Evaluated quality of life	8 mm (3- 15)	87%	nil	In spite of high SFR, less than half the patients benefited subjectively from procedure in terms of improvement in quality of life.
Chung MD, 2008[3]	15* 7 (LC)	RCS with RIRS	1.8 cm*	87%	13%- 1- urinoma, 1- prolonged leak from nephrostomy site	Recurrence 13% at 63 days. SFR and complications higher for PCNL (87 vs. 67%) and (13 vs. 0)

(N- number of patients; SFR- stone-free rate; RCS- retrospective comparative study; CS- case study

* - overall including all LC renal calculi

** -0 included 12 patients with calyceal diverticulum

# - article in Czech)

## LONG-TERM OUTCOME AND STONE RECURRENCE AFTER PERCUTANEOUS NEPHROLITHOTOMY

Residual stone fragments after PCNL confer increased risk of future stone events.[26] Even when a stone-free status is achieved after PCNL, the underlying metabolic abnormalities remain.[7] Comprehensive metabolic evaluation and aggressive medical management can control active stone formation and growth in patients with or without residual stone fragments after PCNL. Kang *et al*., found that selective medical therapy significantly decreased stone formation in stone-free and residual fragment groups after PCNL. Hence, they recommended medical management following PCNL without regard to stone-free status.[27]

Krambeck *et al*., recently published an article on long-term outcome following PCNL.[26] At 19 years follow-up, the stone recurrences were less frequent following PCNL compared to SWL (36.8% vs. 53.5%). PCNL was not associated with development of adverse medical events (new onset renal failure, diabetes mellitus and hypertension) compared with SWL and conservatively managed stone cases.

## RESULTS OF PERCUTANEOUS NEPHROLI-THOTOMY FOR LOWER-POLE CALYCEAL STONE MANAGEMENT

In 1989, McDougal *et al*., were the first to compare the outcome of PCNL with SWL for LC calculi. They noted that PCNL was associated with higher stone-free rates than SWL (86.2 % vs. 54.3%).[7] Similar findings were noted by Netto *et al*., in their retrospective study comparing the outcome of 23 patients treated by PCNL with that of 24 patients treated by SWL.[28] However, since ESWL is a noninvasive procedure without the need for routine anesthesia and hospitalization, and with prompt return of the patient to a normal life they considered it to be the method of choice for treating LC stones less than 2 cm in diameter.

Later on, in 1994, Lingeman *et al*., reported meta-analysis of four series published on PCNL and 13 studies on SWL for LC calculi.[7] They found that overall stone-free rates after SWL were 59.2% and after PCNL were 90%. Among stones of 10 to 20 mm, the stone-free rates were 56% for SWL compared to 89 % for PCNL. On logistic regression analysis, they found that stone size did not affect the stone-free status amongst patients treated by PCNL. In their personal experience of 32 patients with LC calculi treated by PCNL, they had 100% stone-free rates. Because of the significantly greater efficacy of PCNL for LC calculi, particularly stones larger than 10 mm in diameter, authors questioned the appropriateness of SWL as an initial therapy for virtually all LC calculi. Based on their findings, they recommended PCNL as an initial approach to treat these stones. Cass AS reviewed published series of PCNL for lower pole nephrolithiasis and found that the stone-free rate was 70.5-100%, repeat treatment rates were 4-62.5%, the complication rates were 13-38%, and the hospital stay was 3.1 to 6.1 days.[29]

Based on these findings, Albala *et al*., convened a multicentric lower-pole study group (Lower pole study 1) for a prospective multicentric randomized trial comparing the outcome of PCNL vs. SWL in treatment of < 3 cm LC calculi.[30] This study revealed that stone free rates for PCNL were significantly better than for SWL (95% vs. 37%). Morbidity was low overall and did not differ significantly between the groups. The stone-free rates of SWL were only acceptable for stones < 10 mm (63%). Due to this high degree of efficacy and acceptable low morbidity, the lower pole study Group 1 recommended PCNL as an initial modality for treating calculi > 10 mm size.[30] Similar results were observed by other authors in the literature [Table 1].[31,32]

Current flexible ureteroscopes, intracorporeal lithotripsy devices and stone retrieval technology allow for the treatment of calculi located throughout the intra-renal collecting system. Although, difficulty in accessing lower-pole calculi, especially when the holmium laser fiber is utilized, may be encountered, RIRS is associated with 85% stone-free rates as assessed by intravenous pyelography or computerized tomography scan performed at three months.[33] Chung BI compared outcome of PCNL and Ureterorenoscopy (URS) for medium-sized renal calculi (1-2cm).[34] Out of 15 patients who underwent PCNL, seven had lower pole calculi. There were four patients with lower-pole calculi among 12 patients undergoing URS. The authors noted that overall stone-free rate with PCNL was 87% and that for URS was 67% as judged by postoperative KUB imaging. They found that the stone-free rates and complication rates for PCNL are higher, but the differences were not statistically significant. However, cost and durability of flexible ureteroscope still remains an important issue. Till date, there is no randomized control trial evaluating the efficacy of PCNL vs. RIRS for managing renal calculi 1-2 cm in maximum dimension.

## TREND IN MANAGEMENT OF LOWER CALYCEAL CALCULI

In a survey of American urologists conducted by Gerber *et al*., in 2003, two-thirds of the urologists preferred SWL for treating LC calculi of 1-2 cm size. PCNL was preferred by only 30% of urologists at that time.[1] However, approximately five years later, in a survey done by Bandi *et al*., the proportion of urologists preferring PCNL increased and more urologists preferred PCNL to SWL for managing LC calculi [Table 2].[1,2,41] PCNL is the most preferred modality for treating LC calculi with unfavorable anatomy in view of limited clearance of fragments after SWL.

**Table 2:** Trend in management of lower calyceal calculi

Authors, year of study	N	LC stone size	Modality of treatment			Comments
				
			SWL	RIRS	PCNL	
Gerber GS, 2003[1]	205	1-2 cm	65%	5%	30%	First and only survey to know the practice pattern in managing lower pole calyceal stones
Skenazy J, 2005[41]	85	1.5 cm with unfavorable anatomy	20%	12%	68%*	Urologists with > 50% managed care practice are more likely to select PCNL than their counterparts (91% vs. 65%)
						Metropolitan urologists more likely to select PCNL (82% vs. 43%)
Bandi G, 2008[2]	167	1-2 cm symptomatic calculi	43% (29% with stent)	8%	48%	Fellowship trained Endourologist and academic urologist are more likely to recommend PCNL

## LIMITATIONS TO WIDESPREAD ACCEPTANCE OF PERCUTANEOUS NEPHROLITHOTOMY

Although PCNL has high therapeutic success rates independent of stone size, the invasiveness and technically demanding nature limits its use. Similarly, it is perceived to be associated with major complications increasing the patient's morbidity.

### Learning curve

PCNL is currently the most complicated stone surgery technique to teach. The steep learning curve is mainly related to obtaining renal access. A resident has to perform about 24 PCNL procedures to obtain a good proficiency during the residency period. Competence at performing PCNL is reached after 60 cases and excellence is obtained at >100 cases.[13] Similar findings were observed by Allen *et al*.[34]

### Complications

In 1993, Chibber PJ published his experience with 878 patients undergoing PCNL for large and staghorn calculi.[35] Although blood transfusion rate was 12%, only 0.7% patients needed angiography and embolization. The incidence of other complications was very low (urinary tract infection 1.4%; prolonged leak from nephrostomy site 1.3%, hydrothorax 0.5%, bowel fistula 0.1%). In a recently published large series of 1338 patients undergoing PCNL from Canada, the incidence of major complications was similarly low at 3.7%.[36] Tefekli *et al*., classified complications in PCNL according to the modified Clavein grading system and found that most of the complications were Grade 1 or 2.[37] Grade 3a complications (complications requiring surgical, endoscopic, or radiologic intervention without anesthesia) were seen in 6.6% patients and Grade 3b (complications requiring surgical, endoscopic, or radiologic intervention under anesthesia) occurred only in 2.8% patients. Life-threatening complications (Grade 4) occurred in only 1.4% patients and mortality was observed in 0.1% cases.

In the current literature, most of the complications are clinically insignificant bleeding or fever. Significant bleeding is reported in < 8%. Conservative treatment is successful in most cases; however, a 5-18% blood transfusion rate is reported in the literature. In a recent review, the frequency of major complications was 0.9-4.7% for septicemia and 0.6-1.4 % for renal hemorrhage requiring intervention. Access-related complications like pleural and colonic injury were also rare ranging between 2.3-3.1% and 0.2-0.8% respectively.[38]

## CONCLUSIONS

PCNL is a highly effective procedure that may be performed in a diverse group of patients with consistently high stone-free rates when compared with SWL or RIRS. The results also do not depend on anatomic factors and stone size. It is associated with low morbidity in experienced hands. Today in the era of evidence-based medicine, patients should be informed about the available modalities of treatment and their efficacy and safety. Higher stone-free rates associated with PCNL should be stressed while discussing the treatment options with the patients. The appropriate procedure in a given patient should be weighed on a case-by-case basis.
